# Cutaneous neurofibromas: patients’ medical burden, current management and therapeutic expectations: results from an online European patient community survey

**DOI:** 10.1186/s13023-019-1265-4

**Published:** 2019-12-04

**Authors:** Marlene Guiraud, Athmane Bouroubi, Roxane Beauchamp, Arnaud Bocquet, Jean-Marc Grégoire, Isabelle Rauly-Lestienne, Ignacio Blanco, Pierre Wolkenstein, Anne-Marie Schmitt

**Affiliations:** 1 Institut de Recherche Pierre Fabre, Centre de Recherche et Développement, Dermatology Innovation Unit, 3 avenue Hubert Curien – BP 13562 31035, Toulouse Cedex 1, France; 2 Genetic Counseling and Clinical Genetics Program, Laboratori Clínic Metropolitana Nord, Hospital Germans Tias, 08916 Badalona, Spain; 3 CHU Henri Mondor, 94010 Créteil, France

**Keywords:** Neurofibromatosis type 1, Cutaneous neurofibromas, Patient community survey, Quality of life, Future medication, Real-world experience

## Abstract

**Background:**

Neurofibromatosis type 1 is an inherited condition with variable phenotypic expression and a high medical and social burden.

The objectives of this patient survey were to better understand the real-world experiences of patients living with cutaneous neurofibromas (cNF), to perceive their satisfaction and feelings about cNF current management (only laser and surgery are currently available), and to highlight their expectations of new therapeutic modalities.

**Results:**

One hundred seventy patients from 4 European countries took part in the study, 65% (*n* = 110) were women and mean age was 39 years old. 96% (*n* = 164) of respondents have cNF on visible parts of the body and the survey confirmed that total number of cNF and visibility increase with age.

Patients reported that cNF mainly impacts everyday mood, general daily life and social life. The visibility of cNF had a higher impact than their number.

92% (*n* = 156) of patients have a regular and multidisciplinary medical follow-up. The dermatologist is one of the most consulted healthcare professionals.

76% (*n* = 130) of respondents have treated their cNF: 65% (*n* = 111) had surgery and 38% (*n* = 64) had multiple laser sessions. Frequency of operations and regrowth of cNF were the two most unsatisfactory aspects with both treatments for patients. Indeed, after removal, new cNF appear in more than 75% (*n* = 128) of cases.

As a future treatment, patients expected a topical (30%, *n* = 51) or oral medication (29%, *n* = 50). Around 2 out of 3 patients would agree to take it at least once a day or more for life but they would like a well-tolerated treatment.

According to patients, the most important effectiveness criteria of a new treatment are to block cNF growth and reduce their number. 70% (*n* = 119) of patients would consider a future treatment moderately effective to very effective if it could clear 30% of cNF.

**Conclusions:**

This first cNF European patient community survey confirmed that the visible stigma and unaesthetic aspect of cNF have an important impact on patients’ quality of life. The survey highlighted that patients were not entirely satisfied with the actual surgery and laser treatments and revealed their clear and realistic expectations for future treatment of cNF.

## Background

Neurofibromatosis type 1 (NF1), also called von Recklinghausen’s disease, is a genetic disorder caused by a mutation in or a deletion of the neurofibromin gene, which is characterized by the development of multiple tumours on nerves throughout the body. NF1 has a variable phenotypic expression, including cutaneous, neurological, skeletal and neoplastic complications, with an unpredictable rate of progression [1].

The average prevalence of NF1 is one in 2500 to 3000, so that about 2.5 million people worldwide are affected. In 50% of affected individuals, NF1 results from spontaneous mutations of the gene that occur for unknown reasons [2]. NF1 affects males and females equally and all ethnicities [3].

Clinical diagnosis of the disease is based on the presence of café-au-lait macules, axillary or inguinal freckling, Lisch nodules, optic pathway gliomas, bony dysplasia, and neurofibromas. Neurofibromas can be classified according to their anatomical location: plexiform, intraneural, subcutaneous and cutaneous [4, 5]. Plexiform neurofibromas are congenital deep tumours involving nerve plexus below the dermis with a risk of sarcomatous transformation; intraneural neurofibromas grow in the peripheral nerves; subcutaneous neurofibromas occur along the peripheral nerves beneath the skin; and cutaneous neurofibromas ([cNF] also called dermal neurofibromas) are benign tumours that grow from small nerves present in and/or just under the skin. Clinically they look like well-defined cutaneous lesions, small bumps typically beginning around the time of puberty [6, 7]. cNF affect 99% of NF1 patients, starting from puberty and increasing in size and number with age [1, 8].

The natural history of cNF is still poorly understood due to an extensive interpatient and intrapatient heterogeneity in cNF presentation, degree of severity and behavior over time described extensively in the literature [9]. Although these lesions are not life-threatening, they are known to dramatically affect the quality of life of patients due to the visibility of the lesions and stigmatization [1, 10–12].

Considering the major impact cNF has on patients’ quality of life, the medical need is huge. Unfortunately, there is no treatment approved to cure cNF, patients are currently offered surgical removal or physical destruction using laser or electrodessication [7, 9]. In parallel, notable therapeutic advances have been made for plexiform neurofibromas and recent clinical trials evaluating MEK inhibitors have shown promising results [13].

As a growing influence in their own care pathway and treatment, patients play an important role in the management of their disease, the choice of treatment and product compliance, and consequently can influence the success or failure of therapeutic strategies.

In this regard, improving the lives of patients requires a deep understanding of their medical condition, feeling of disease burden, needs and priorities. Thus, an online patient survey was conducted in adult patients with cNF in 4 European countries with three main objectives: 1) to better understand the real-world experiences of patients living with cNF, 2) to perceive patients’ satisfaction and feelings with cNF current management and 3) to highlight patients’ expectations related to a new treatment.

## Methods and design

The study was conducted in collaboration with *Carenity*, a leading online patient community platform in Europe with more than 1200 communities and 250,000 patients registered. This online patient platform provides an opportunity for patients and caregivers to share their disease experience, to get informed and contribute to medical research by participating in surveys.

The first step of the study involved setting up a new community comprising adult patients with cNF due to NF1. One platform was created for each country (France, United Kingdom, Spain, and Germany), so that information was always displayed in the local language. Patients were informed about the existence of the community either via the patients’ association or via websites such as facebook advertisements. The platform was free, voluntary, and anonymous.

Once registered, the patients were informed and consented to the collection, handling and keeping of their personal data, in particular of their health data. Carenity informs members of the purpose of the collection and processing of their personal data in a full and transparent way.

The second step of the study involved organizing a forum discussion animated in the French and Spanish online platforms to collect patients’ verbatim responses and identify the topics of interest for the patients’ survey. For that purpose, four main specific discussions were managed by the community managers related to cNF description, their impact on quality of life, satisfaction of patients with laser and surgery treatments and expectation related to future treatments. Twenty-three patients participated; all the comments posted were extracted then selected and grouped by themes. Pertinent comments were considered to enrich the patient questionnaire.

The survey questionnaire, designed in English, was made of 37 closed-ended questions and 5 open-ended questions on the following domains: patients’ profile, living with cNF, patients’ satisfaction with cNF management and patients’ expectations in terms of future treatments (see Additional file 1). Three additional questions were slipped into the questionnaire as a comprehension test to ensure that patients were able to answer every kind of question (checking test, ranking test, and table test).

The survey questionnaire was validated by two medical experts of neurofibromatosis and finally reviewed by one patient representative to confirm the questionnaire was understandable to a lay person. Once the survey was validated, it was translated into local language by native speaking linguists. Following review and validation of the translated questionnaire, a final review was performed by each community manager at Carenity to ensure the language was accessible to a lay audience. The translated questionnaire was posted on the 4 platforms and the community members were invited to answer online, either when connected to the platform or by weekly emails. The study was conducted between May 2018 and June 2018.

The patients were enrolled in the study only if they fulfilled the following inclusion criteria: 1) adult patients who declared to be affected by NF1, 2) adult patients with cNF (several pictures of cNF were displayed in the questionnaire), and 3) adult patients living in France, Germany, Spain or the United Kingdom.

### Data analysis

Only the completed questionnaires were considered for the analysis. Participants data were kept pseudonymised during the analysis and only aggregated data were presented in the results.

Excel 2013® was used to perform the different analyses and R studio (v3.5.0) was used to perform multiple correspondence analysis and statistical analysis. Overall analysis and per country analysis were performed for every question, multivariate analysis was performed to take into account several items and questions at the same time.

For the purpose of the study, two scores were set up and validated with the Advisory Board (comprised of the two medical experts in neurofibromatosis, Pr Pierre Wolkenstein and Pr Ignacio Blanco [who also validated the survey questionnaire]) to characterize disease severity and visibility and to allow the analysis with multiple factors that may be influenced by the severity and visibility of the cNF. Severity grade (S Grade) was based on the total number of cNF on the whole body and Visibility grade (V Grade) based on the number of cNF present on visible parts of the body (face, hands, forearm, and neck). The classification was defined with 5 grades:

**Table:** 

S and V Grade	1	2	3	4	5
Number of cNF	≤ 10	11–50	51–100	101–500	> 500

The sample size objective was 150 completed questionnaires with a well-balanced participation of each country to allow descriptive overall and per country analysis.

## Results

### Patients’ profile

A total of 219 questionnaires were completed: 28 were excluded due to the inclusion criteria not being met (8 patients were not affected by NF1, 19 did not have cNF and 1 patient was younger than 18 years old (y/o)) and 21 did not pass the quality control (inconsistency verbatim or anti-chronological date found in the questionnaire) leaving 170 questionnaires that were completed and selected for the study analysis.

Among the 170 participants, 44% (*n* = 75) lived in France, 19% (*n* = 32) lived in Spain, 19% (*n* = 33) lived in Germany, and 18% (*n* = 30) lived in the United Kingdom with overall participants comprising 65% (*n* = 110) women and 35% (*n* = 60) men. The mean age was 39 y/o with a majority of patients (62% [*n* = 106]) in the 26–45 y/o range. More than half of patients reported having children (55% [*n* = 94]), with more than half of them having 2 children (54% [*n* = 51]).

61% (*n* = 104) patients reported to have learning difficulties cause by NF1; behavioral manifestations such as attention-deficit/hyperactivity disorder, are indeed a complication of the disease well-described in the literature, nevertheless 78% (*n* = 133) of respondents successfully completed the comprehension test (Table 1).

**Table 1:** Characteristics of patients - overall and per country

	All patients (*n* = 170)	France (*n* = 75; 44%)	Spain (*n* = 32; 19%)	UK (*n* = 30; 18%)	Germany (*n* = 33; 19%)
Age, mean [min-max]	39 [18–79]	41 [18–79]	40 [24–73]	35 [19–68]	40 [19–68]
Repartition by class age n (%)					
18–25 y/o	20 (12)	8 (11)	2 (6)	5 (17)	5 (15)
26–45 y/o	106 (62)	47 (63)	22 (69)	20 (67)	17 (52)
46–65 y/o	35 (21)	15 (20)	6 (19)	4 (13)	10 (30)
> 65 y/o	9 (5)	5 (7)	2 (6)	1 (3)	1 (3)
Gender, woman n (%)	110 (65)	55 (73)	13 (41)	19 (63)	23 (70)
Family situation: % with children (mean number of children)	55 (1.9)	53 (2.0)	59 (1.7)	57 (1.9)	55 (1.8)
Learning difficulties caused by NF1 n (%)	104 (61)	53 (71)	20 (63)	14 (47)	17 (52)

Description of the patients’ characteristics overall and for each country: mean age, repartition by class age, gender, family situation with % of patients with children, mean number of children and learning difficulties caused by NF1

### Disease characteristics

On average, the patients declared that the first cNF appeared at 14 y/o. The most frequently reported circumstances where patients noticed the appearance or growth of cNF were pregnancy, puberty, and stress (Table 2).

**Table 2:** Circumstances around the appearance or growth of cNF - overall and per country

Circumstances n (%)	All patients (*n* = 170)	France (*n* = 75; 44%)	Spain (*n* = 32; 19%)	UK (*n* = 30; 18%)	Germany (*n* = 33; 19%)
Can appear without any specific reasons	84 (49)	49 (65)	10 (31)	8 (27)	17 (52)
Puberty	77 (45)	34 (45)	15 (47)	13 (43)	15 (46)
Stress	75 (44)	35 (47)	8 (25)	17 (57)	15 (46)
Pregnancy^a^	47 (43)	28 (51)	3 (23)	9 (47)	7 (30)
Sun exposure	35 (21)	10 (13)	7 (22)	11 (37)	7 (21)
Hormonal treatments^a^	29 (26)	9 (16)	4 (31)	5 (26)	11 (48)
Piercing	5 (3)	0 (0)	1 (3)	2 (7)	2 (6)
Skin incision or injury	4 (2)	1 (1)	0 (0)	1 (3)	2 (6)
Other	5 (3)	5 (7)	0 (0)	0 (0)	0 (0)

Description of the circumstances noticed by the patients around the appearance or growth of cNF overall and for each country

^a^ % of female patients reported

96% (*n* = 164) of respondents declared they have cNF on visible parts of the body (i.e. on the face, neck, hands, forearm).

**Fig. 1:**
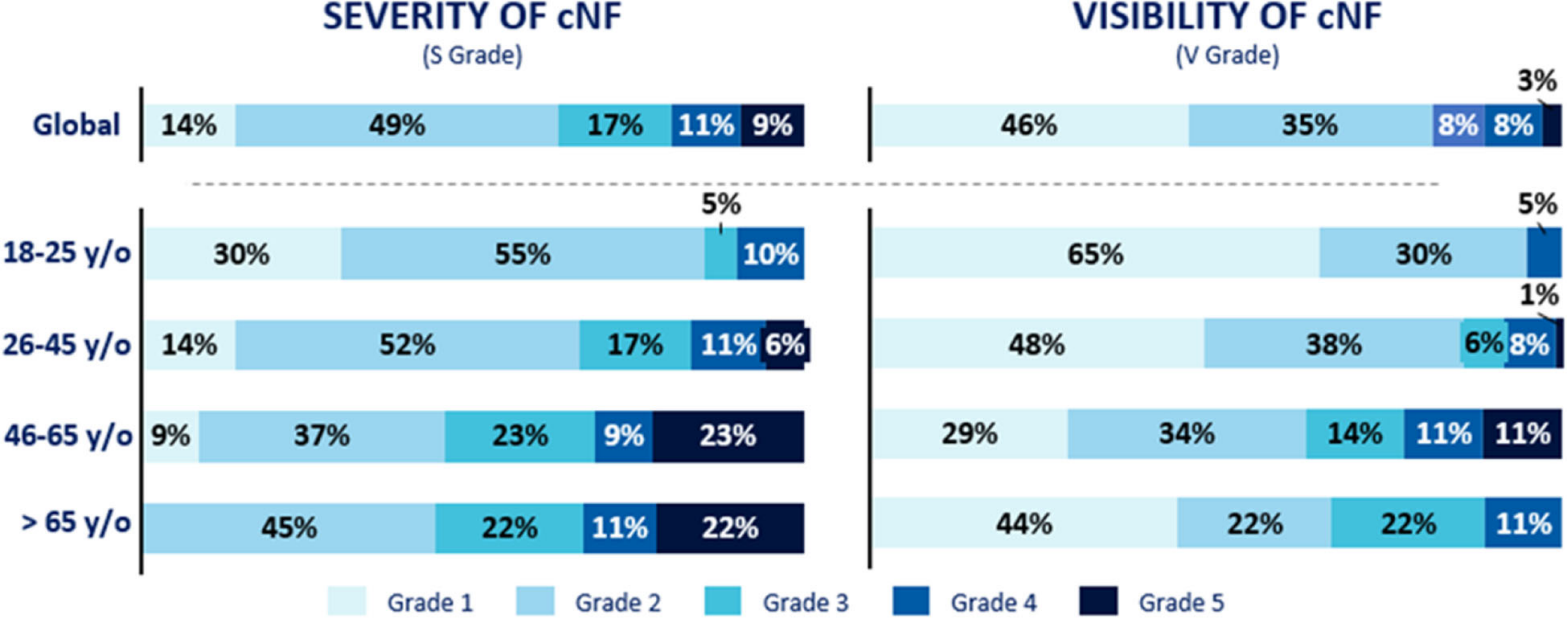
Severity and visibility of cNF depending on the age of patients. Severity grade (total number of cNF on the whole body) and visibility grade (number of cNF present on visible parts of the body) presented globally and by patient age from grade 1 (≤ 10 cNF) to grade 5 (> 500 cNF)

Total number of cNF and visibility increase with age: 15% of respondents aged between 18 and 25 y/o have a severity grade of 3 and higher vs 56% in the older population > 65 y/o and 5% vs 33% for a visibility grade of 3 or higher (Fig. 1).

### Patients living with cNF

The main inconvenience caused by cNF and reported by patients was the unaesthetic aspect and visibility of the tumours followed by the possible evolution of the disease severity (mean rank: 3.5 and 3.6 respectively).

**Fig. 2:**
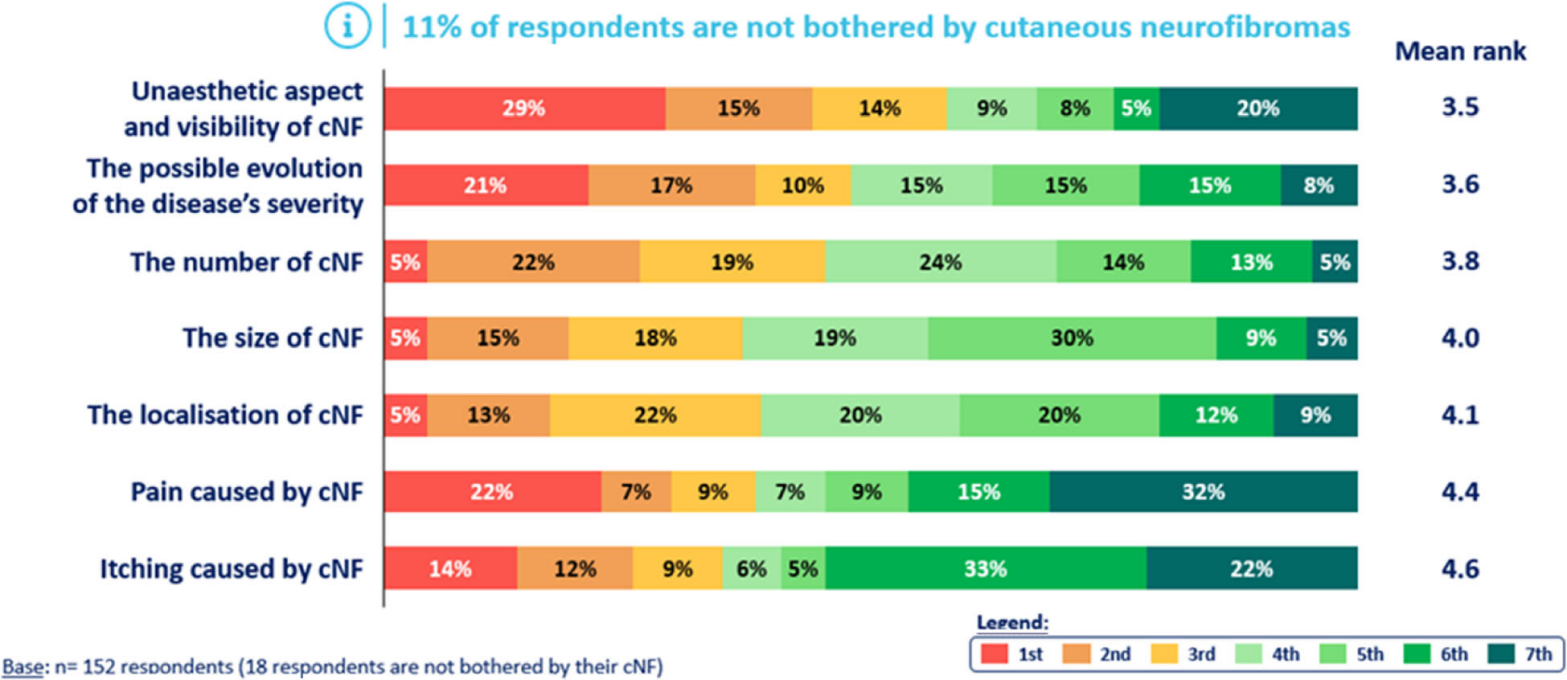
Inconveniences caused by cNF as reported by the patients. Description of the main inconveniences caused by cNF to the patients ranked from the most bothersome aspect (rank 1) to the least bothersome (rank 7). The inconveniences are filed according to the mean rank

Overall, pain and itching caused by cNF were the least bothersome (mean rank: 4.4 and 4.6 respectively) but begin to be burdensome when they are felt, 22% (*n* = 34) and 14% (*n* = 21) of concerned patients ranked them 1st (Fig. 2).

Only 11% (*n* = 18) of the patients reported not to be bothered by their neurofibromas; in those respondents, the percentage of men is higher (44% vs 34%) and severity and visibility grade are lower (e.g. all patients who were not bothered by their neurofibromas have visibility grade 1 or 2, whilst 79% of patients who were bothered by their neurofibromas have visibility grade 1 or 2 and 21% have visibility grade > 2).

Men and women rank inconveniences in the same way, except for the size of cNF which is more bothersome for men (mean rank: 3.5) than for women (mean rank: 4.3).

As expected, the patients reported that cNF affects several aspects of their quality of life, mainly everyday mood (mean: 5.6/10, 10 = considerable impact), daily life in general (5.1), and social life (4.8).

Patients’ quality of life is impacted more if cNF are visible and have appeared a long time ago.

The more severe or more visible the cNF are, the more it impacts the quality of life of the patients in all the domains: family life, professional life, social life, private life, everyday mood and daily life in general (Table 3).

**Table 3:** Impact of cNF on quality of life depending of cNF severity and visibility

Mean	Average impact	Family life	Professional life	Money spent for cNF	Desire to have children	Private life	Social life	Daily life in general	Everyday mood
S Grade 1 (*n* = 24)	4.3	3.8	3.7	4.2	4.9	4.3	4.4	4.4	4.9
S Grade 2 (*n* = 83)	4.3	3.2	3.7	3.6	4.2	4.8	4.9	4.9	5.4
S Grade 3 (*n* = 29)	3.7	3.3	2.7	2.7	4.3	3.4	3.4	4.4	4.8
S Grade 4–5 (*n* = 34)	5.7	4.8	4.4	4.6	5.3	6.2	6.3	6.4	7.1
V Grade 1 (*n* = 78)	4.1	3.0	3.3	3.4	4.3	4.6	4.4	4.5	5.1
V Grade 2 (*n* = 60)	4.4	3.7	3.7	3.6	4.7	4.4	4.8	5.0	5.5
V Grade 3 (*n* = 13)	5.0	4.5	4.8	3.8	3.3	5.0	5.0	6.2	5.9
V Grade 4–5 (*n* = 19)	6.1	5.5	4.6	5.2	5.6	6.4	6.7	6.8	7.5

Score from 0 to 10, 0 = no negative impact and 10 = considerable negative impact

Description of the impact of cNF on several aspects of quality of life: family life, professional life, economic burden, private life, social life, daily life in general, and everyday mood – by a mean score. Results are presented by severity grade and visibility grade

The impact of cNF increased with age, (average impact 3.6 for 18–25 y/o vs 5.3 for > 65 y/o). The greater impact of cNF with age was especially true for everyday mood, daily life, social life, and private life.

### Medical monitoring

**Fig. 3:**
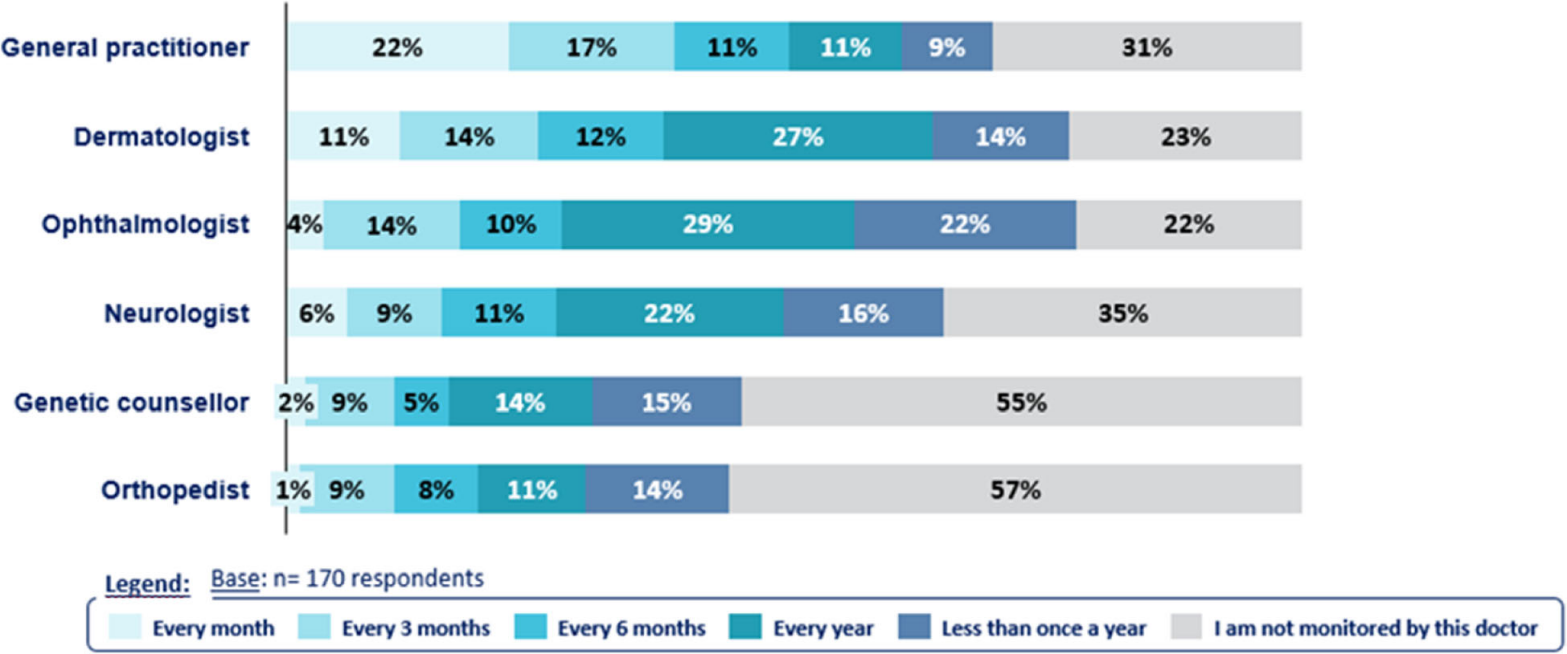
Medical monitoring of NF1 patients. Description of the medical monitoring of patients, which is broken down by physician specialty and frequency of consultation

Most of the patients reported to have a regular and multidisciplinary medical follow-up. Overall, 92% (*n* = 156) of patients consulted one healthcare professional regularly. Many healthcare professionals are involved in NF1 monitoring due to complexity of the disease and multiple potential complications. On average, patients reported to be followed by 3.8 doctors (3.4 in France, 3.8 in Germany, 4.3 in Spain, and 4.4 in UK). The general practitioner is consulted the most often (22%, *n* = 37 every month), dermatologist is consulted by about 77% (*n* = 131) of the patients, a little less in Germany (64%, *n* = 21)) (Fig. 3).

More than half of respondents (54%, *n* = 91) are regularly seen by a neurofibromatosis specialist, these patients are slightly younger than the ones who do not see specialists (37.6 y/o vs 41.2 y/o). A greater proportion of UK patients accessed specialized consultation (67%, *n* = 20).

The younger respondents had more frequent consultation with several healthcare professionals than the older ones, which is in-line with recent epidemiology studies highlighting that diagnosis and monitoring increase in the young population [4].

### Patient’s satisfaction regarding cNF management

Overall, more than 76% (*n* = 130) of respondents have treated their cNF with either a CO_2_ laser or surgical intervention. Surgery is the most prescribed treatment (65%, *n* = 111), whereas 38% (*n* = 64) had laser sessions and 26% (*n* = 45) experienced both techniques. A greater proportion of respondents in Germany (33%), Spain (41%), and France (43%) received laser treatment for their cNF compared to only 27% of UK respondents.

The type of treatment depends on the country: UK respondents have limited access to treatments, about 40% (*n* = 12) of the patients did not treat their cNF.

The respondents who did not have laser or surgery are younger than the ones who already experienced laser or surgery (mean age: 34 vs 41 y/o). Treatment with laser and/or surgery increased with severity and visibility of cNF: from severity or visibility grade 3, more than 50% of patients have been treated by laser and more than 75% by surgery.

Whether by surgery or laser, visible cNF are removed as priority; approximately 50% of patients had only visible cNF removed.

**Fig. 4:**
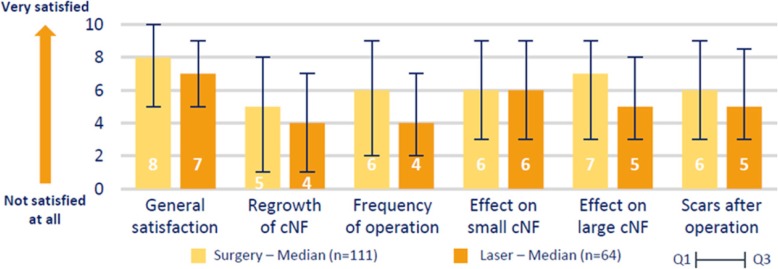
Satisfaction with surgery and laser. Presentation of patients’ level of satisfaction towards surgery and laser on several aspects: general satisfaction, regrowth of cNF after procedure, frequency of operation, effect on small cNF and appearance of scars after procedure. The results are presented as the median score and Q1/Q3 quartiles

Overall, respondents were quite satisfied with surgery and laser treatments (median rank for general satisfaction: 8 and 7) and patients are slightly more satisfied with surgery than the laser. The frequency of surgery and regrowth of cNF are the two most unsatisfactory aspects experienced by patients with both treatments. Indeed, after removal, new cNF appear in more than 75% of cases (Fig. 4).

French respondents were the least satisfied with laser sessions, especially regarding the regrowth of cNF (median rank: 3), accessibility of laser sessions under general anesthesia [3] and frequency of laser sessions [4].

As for the surgery, French and Spanish respondents were the least satisfied, especially due to regrowth of the lesions (median rank: 4 for both countries), frequency of surgeries (5 for both countries) and number of cNF removed (4.5 for France and 5 for Spain), whereas German respondents were the most satisfied in general (median rank: 9).

Overall, only 9% of respondents had more than 75% of their cNF removed by surgeries or laser session. Approximately half of the respondents had ≤25% of their cNF removed by surgeries or laser.

In addition to invasive treatment, a quarter of respondents reported that they already took other medications or used alternative medicines to manage their cNF, nevertheless it strongly depends on the country since in the UK 53% (*n* = 16) patients reported to take one or other, while in France it is only 8% (*n* = 6). Respondents mainly mention creams, acupuncture, physiotherapy, and antidepressants as medication or supportive care to reduce pain and itching or more globally to manage their cNF.

### Patient’s expectation towards potential future treatments

Patient’s expectations towards future treatments were equally distributed between topical form (cream or ointment for 30% of patients), oral medication (pill or capsule for 29% of patients) or implant, whereas injection was the least popular galenic form, except for the UK patients who ranked this formulation as second highest (mean rank: 2.4).

The age of the respondents did not strongly influence the choice of the galenic form of future treatment, nevertheless implants were ranked higher by the oldest (> 45 y/o) and oral formulation ranked higher by the youngest patients (≤ 45 y/o).

cNF visibility did not seem to have a strong impact on the ranking of the galenic form of future treatment. Nevertheless, implants were considered the best form of future treatment for patients with a high visibility cNF grade (3 and 4-5) compared to oral formulation for the other patients (grades 1 and 2).

Sixty percent (*n* = 102) of respondents declared that they would be ready to take an effective treatment for life, and greater for patients with higher severity grade cNF: 33% (*n* = 8) of grade 1, 54% (*n* = 45) of grade 2, 69% (*n* = 20) of grade 3 and 85% (*n* = 29) of grade 4/5.

Around 2 out of 3 patients would agree to take an oral treatment or apply a cream at least once a day or more, and greater for the patients with high severity grade (61% of grade 1, 81% of patients with grade 4/5)*.*

**Fig. 5:**
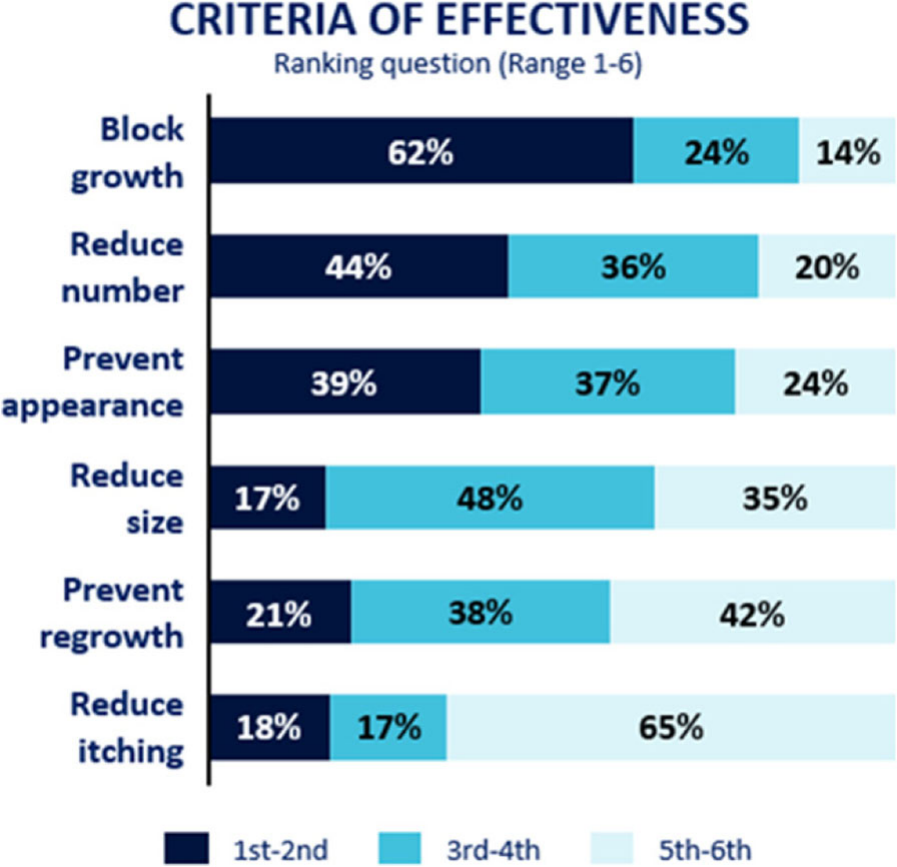
Effectiveness criteria for a future treatment. Presentation of effectiveness criteria for a future treatment from the most important (rank 1st-2nd) to the least important (rank 5th–6th)

According to patients, the most important effectiveness criteria of a new treatment were to block cNF growth (1st or 2nd choice for 62%, *n* = 105)) and to reduce their number (1st or 2nd choice for 44%, *n* = 74) (Fig. 5).

When expressed by the number of cleared cNF, 70% (*n* = 119) of patients would consider a future treatment moderately effective to very effective if it could clear 30% of cNF.

Overall, whatever the severity of the disease, the respondents were more likely to agree to take a medication for up to 6 months before seeing a visible effect on their skin (63%, *n* = 107).

**Fig. 6:**
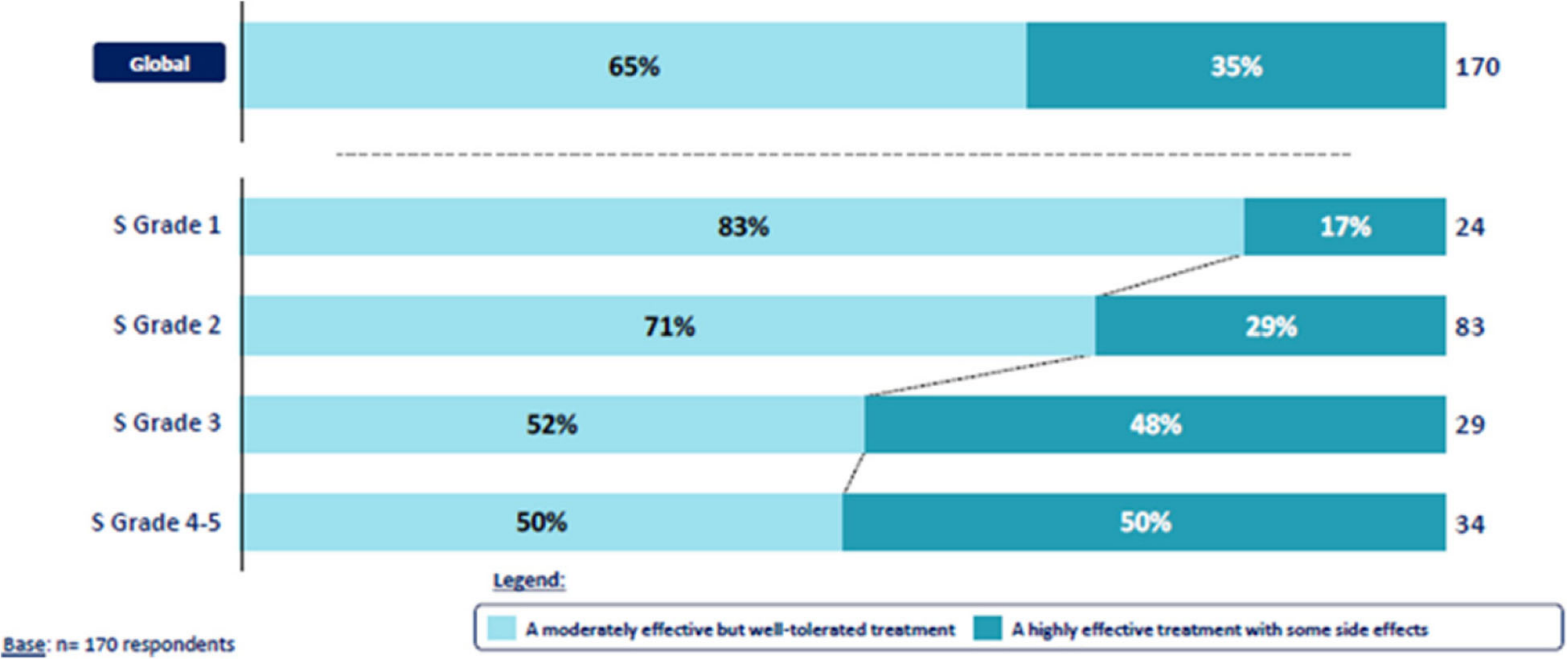
Risk benefit balance depending on the cNF severity. Presentation of the risk benefit balance globally and by cNF severity grade expressed as the % of patients who preferred a moderately effective but well tolerated treatment compared to a highly effective treatment with some side effects

Overall, the patients were keen to have a well-tolerated treatment: 65% (*n* = 111) respondents would prefer a moderately effective but well-tolerated treatment rather than a highly effective treatment with some side effects, nevertheless the most severely affected patients reported that they would be prepared to tolerate some side effects to have a highly effective treatment: only 17% (*n* = 4) of respondents with a severity grade 1 would accept some side effects while 50% (*n* = 17) of respondents with a severity grade 4 or 5 were prepared to accept side effects (Fig. 6).

## Discussion

Patients are increasingly actively involved in health care and patient empowerment in the health system is becoming a reality. A patient-centred approach in the research and development process of new medicines has become an obvious strategy to better understand the medical needs and to ensure relevance and suitability of the treatment under development. Patients and their families can act as advocates for the drugs and help to get drugs to other patients faster. This is even more important in the context of rare diseases.

The present study highlights the real-world experiences of patients living with cutaneous NF. To our knowledge, this is the first time such a survey is performed for this rare condition and the findings provide relevant information on the disease burden and natural history of cNF. In addition, it highlights the target product profile of a topical or an oral treatment that would first block the cNF growth and would be well-tolerated.

Furthermore, the patients mentioned the blocking of the cNF growth followed by the reduction of cNF numbers and thus reduced visibility as the main expected efficacy criteria. 70% of patients would consider a future treatment moderately effective to very effective if it could clear away a minimum of 30% cNF; these results showed that patients are well informed about their genetic condition and the natural history of cNF. They have realistic expectations on treatment, they do not expect a complete clearance of the lesions. This is of particular importance for the development of clinical endpoint to define the relevance of clinically significant effect. When regulatory agencies request complete or near complete clearance of skin lesions to approve new drugs the patient voice should be considered to define the minimal acceptable efficacy level. NF1 is a chronic disease characterized by the appearance of cNF in almost all patients, starting around puberty age and increasing throughout life. Even the natural history of the disease is still poorly understood; almost all the publications on cNF describe a strong correlation of cNF with age and a worsening condition with age due to the increase of number, size and visibility of the cNF [6, 14]. However, it is uncommon to find precise and quantified data supported by a score or a scale. Indeed, both the severity scale of Riccardi and the visibility index of Ablon are validated to characterize the severity of NF1 in its entirety taking into account the multiple potential complications of the disease but these scales do not allow the cNF condition specifically to be characterized.

Using the severity and visibility scores designed for the study, the survey clearly confirmed a strong correlation of the severity and visibility of cNF with age. It also highlighted the strong influence of the severity and visibility on the quality of life of the patient on every domain of the scale.

With regard the patient’s expectation on future treatment for cNF, overall, the results of the study pointed out very realistic demands whatever the galenic form of the treatment, and indicated some significant influence of the severity and visibility of the condition on the patient’s need: patients with the most severe condition would be ready to tolerate additional side effects to have a highly effective treatment, whereas patients with less severe conditions would prefer a moderately effective treatment but well-tolerated. The most severe patients would also be prepared to take a treatment more frequently and over a longer period.

Whatever the cNF severity, blocking cNF growth is the most important effectiveness criterion of a future treatment, nevertheless this criterion is increasingly important with increasing cNF severity; patients with the most severe conditions have less demanding expectations in terms of clearance rate of a new treatment.

So, the severity and visibility scores designed for the study succeeded in characterizing the study population and captured the expectations of patients with different phenotypes. It would be of great interest to develop and validate specific scales for cNF for the purpose of clinical studies and to follow up patients in clinical practice.

As described in the literature and demonstrated in some previous studies [8, 15], our survey confirmed that puberty and pregnancy are significant trigger factors of cNF noticed by patients. More surprisingly, two environmental factors have been associated by the patients with the appearance of growth of cNF: stress and sun exposure (reported by 45 and 20% of patients respectively). This is an unexpected finding since it is not described in the natural history of the disease. This finding should be explored further to assess whether stress prevention and sun protective measures should be considered by doctors in the future. UV-induced DNA damage may be a cause of somatic second-hit mutations and the development of face or neck cNF. It may be worth testing these conditions in preclinical animal models.

Another topic of interest highlighted by the survey is related to pain and itching symptoms caused by the cNF. Overall, these are the least bothersome symptoms (mean rank: 4.4 and 4.6 compared to unaesthetic aspect and visibility rank 3.5) but pain and itching are ranked as most bothersome for 22 and 14% patients respectively. In the literature, few data are available on pain and itching, but some publications report that NF1 is associated with pruritus in about 20% of patients [7, 16] and up to 35% in a French study performed specifically to characterize pruritus in NF1 patients, 18% of whom also had pain accompanying their pruritus [16]. This study highlighted that chronic pruritus can significantly contribute to the quality of life of patients as it frequently affects sleep and is associated with pain. For the patients affected by these symptoms, reducing the itching or the pain is one the most important effectiveness criteria of a future treatment. The study of Khosrotehrani et al. [17] demonstrated that pruritus was mainly localized to one or more cNF, supporting the advantages of a topical treatment to alleviate localized itching.

A large majority of the patients participating in our study mentioned they benefit from a regular and multidisciplinary medical follow-up. More than half of the patients are regularly seen in a specialized medical center and globally dermatologists are one of the most consulted healthcare professionals. Nevertheless, it is true that consultation of healthcare professionals varies vastly from one country to another, for example our study highlights that patients in the UK have more access to specialized consultations compared to other countries and that Spanish patients have more access to dermatologists than German patients.

Access to treatment may also be influenced by the country. Overall more than 75% of patients have treated their cNF by surgery and/or laser. Nevertheless, the type of invasive treatment depends on the country, for example our study highlights that UK respondents have limited access both to laser and surgery and 40% of the population remained untreated.

No publications summarize and compare medical follow-up between European regions and also with the US, which could be of great interest to highlight differences in the cNF management, medical pathway, this may be useful to optimize the conduct of international clinical study for example. This may also reveal different unmet medical needs and diverse expectations regarding future treatment.

It should be noticed that in most European countries, the guidelines for the management of individuals with NF1 recommend that adult patients with NF1 attend the neurofibroma clinic or neurofibroma specialist on an annual basis [18–20].

The respondents to the questionnaires reported more frequent consultation with several doctors, they are certainly more regularly treated with surgery and laser than the overall NF1 population. This may reflect a bias in the patient selection with the online patient community. The respondents of the survey may be more closely monitored and attentive to their disease, which may not be representative of the overall NF1 population.

This web-based patient survey was based on voluntary inscription and participation of the patients. Patient’s data regarding diagnosis, disease description and treatment received were not confirmed by medical practitioners.

In addition, the respondents may not be representative of the global population; it is known from the literature that about 50% of the NF1 population may not be diagnosed and not followed by medical centers. The respondents of this survey may be particularly well monitored and mindful of their disease.

## Conclusions

This study highlights for the first time the real-word experiences of European patients living with cNF and their medical monitoring. The study confirms that the visible stigma and unaesthetic aspect of cNF have an important impact on a patient’s everyday mood and daily life. The majority of patients have regular and multidisciplinary medical monitoring and have already experienced surgery and/or laser treatment. Nevertheless, they are not entirely satisfied by these procedures mainly due to regrowth after the interventions. The study highlights the medical need for future treatments for cNF: a topical or an oral form that would primarily block the cNF growth. A majority of patients would be willing to take it daily, for life, and would like to have a well-tolerated treatment. Thus, this study reinforces the ability of an online real-world study to accurately capture important information on patients own experience with NF1 and realistic treatment expectations.

## Supplementary information

**Additional file 1.** Patient survey for online Carenity patient community ‘Project Neurofibromatosis type 1 with cutaneous neurofibromas’. The survey for neurofibromatosis type 1 patients with cutaneous neurofibromas (cNF) includes 37 closed-ended questions and 5 open-ended questions on the following domains: patients’ profile (age, gender, family situation, learning difficulties, number of cNF, age at time of first cNF, circumstances around cNF appearance), living with cNF (inconveniences caused by cNF, impact on quality of life, medical monitoring, access to specialized medical center), patients’ satisfaction with cNF management (access to and satisfaction with laser and/or surgical treatments, other medications used), and patients’ expectations in terms of future treatments (best galenic form, effectiveness criteria, risk benefit balance, acceptable period of treatment, acceptable frequency of administration). Three additional questions are slipped into the questionnaire as a comprehension test (checking test, ranking test, and table test).

## Data Availability

The datasets used and analysed during the current study are available from the corresponding author on reasonable request.

## Abbreviations

cNF: Cutaneous neurofibromas
NF1: Neurofibromatosis type 1
y/o: Years old

## References

[1] Cannon A, Chen MJ, Liz P, Boyd KP, Theos A, Redden DT, et al. Cutaneous neurofibromas in Neurofibromatosis type I: a quantitative natural history study. Orphanet J Rare Dis. 2018. 10.1186/s13023-018-0772-z. 10.1186/s13023-018-0772-z PMC5803843 29415745

[2] Kallionpää RA, Uusitalo E, Leppävirta J, Pöyhönen M, Peltonen S, Peltonen J (2018). Prevalence of neurofibromatosis type 1 in the Finnish population. Genet Med.

[3] Anderson JL, Gutmann DH (2015). Neurofibromatosis type 1. Handb Clin Neurol.

[4] Jouhilahti EM, Peltonen S, Callens T, Jokinen E, Heape AM, Messiaen L (2011). The development of cutaneous neurofibromas. Am J Pathol.

[5] Gutmann DH, Ferner RE, Listernick RH, Korf BR, Wolters PL, Johnson KJ (2017). Neurofibromatosis type 1. Nat Rev Dis Primers.

[6] Allaway RJ, Gosline SJC, La Rosa S, Knight P, Bakker A, Guinney J (2018). Cutaneous neurofibromas in the genomics era : current understanding and open questions. Br J Cancer.

[7] Ortonne Nicolas, Wolkenstein Pierre, Blakeley Jaishri O., Korf Bruce, Plotkin Scott R., Riccardi Vincent M., Miller Douglas C., Huson Susan, Peltonen Juha, Rosenberg Andrew, Carroll Steven L., Verma Sharad K., Mautner Victor, Upadhyaya Meena, Stemmer-Rachamimov Anat (2018). Cutaneous neurofibromas. Neurology.

[8] Verma SK, Riccardi VM, Plotkin SR, Weinberg H, Anderson RR, Blakeley JO (2018). Consideration for development of therapies for cutaneous neurofibroma. Neurology.

[9] Cannon A, Jurnagin K, Korf B, Widemann BC, Casey D, Ko HS (2018). Clinical trial design for cutaneous neurofibromas. Neurology.

[10] Wolkenstein P, Zeller J, Revuz J, Ecosse E, Leplège A (2001). Quality-of-life impairment in neurofibromatosis type 1: a cross-sectional study of 128 cases. Arch Dermatol.

[11] Kodra Y, Giustini S, Divona L, Porciello R, Calvieri S, Wolkenstein P (2009). Health-related quality of life in patients with neurofibromatosis type 1: a survey of 129 Italian patients. Dermatology.

[12] Page PZ, Page GP, Ecosse E, Korf BR, Leplege A, Wolkenstein P (2006). Impact of neurofibromatosis 1 on quality of life: a cross-sectional study of 176 American cases. Am J Med Genet A.

[13] Dombi E, Baldwin A, Marcus LJ, Fisher MJ, Weiss B, Kim A (2016). Activity of Selumetinib in Neurofibromatosis type 1-related plexiform neurofibromas. N Engl J Med.

[14] Ehara Y, Yamamoto O, Kosaki K, Yoshida Y (2018). Natural course and characteristics of cutaneous neurofibromas in neurofibromatosis 1. J Dermatol.

[15] Brosseau JP, Pichard DC, Leguis EH, Wolkenstein P, Lavker R, Blakeley JO (2018). The biology of cutaneous neurofibromas: consensus recommendations for setting research priorities. Neurology.

[16] Brenaut E, Nizery-Guermeur C, Audebert-Bellanger S, Ferkal S, Wolkenstein P, Misery L (2016). Clinical characteristics of pruritus in Neurofibromatosis 1. Acta Derm Venereol.

[17] Khosrotehrani K, Bastuji-Garin S, Riccardi VM, Friedman JM, Wolkenstein P (2005). Subcutaneous neurofibromas are associated with mortality in neurofibromatosis 1: a cohort study of 703 patients. Am J Med Genet A.

[18] Protocole National de Diagnostic et de Soins (PNDS) Neurofibromatose 1 Décembre 2016. https://www.has-sante.fr/portail/upload/docs/application/pdf/2016-12/pnds_-_neurofibromatose_de_type_1.pdf. Last accessed 11 Jul 2019.

[19] Ferner RE, Huson SM, Thomas N, Moss C, Willshaw H, Evans DG (2007). Guidelines for the diagnosis and management of individuals with neurofibromatosis 1. J Med Genet.

[20] Friedman JM. Neurofibromatosis 1. 1998 Oct 2 [Updated 2018 May 17]. In: Adam MP, Ardinger HH, Pagon RA, et al., editors. GeneReviews® [Internet]. Seattle: University of Washington; 1993-2019.

