# Supplementary material for: Cutaneous neurofibromas: patients’ medical burden, current management and therapeutic expectations: results from an online European patient community survey
Source: Orphanet J Rare Dis. 2019 Dec 4;14:286. doi: 10.1186/s13023-019-1265-4 (PMC6894276; doi:10.1186/s13023-019-1265-4)
Supplement: Supplementary file 1 — Additional file 1. Patient survey for online Carenity patient community ‘Project Neurofibromatosis type 1 with cutaneous neurofibromas’. The survey for neurofibromatosis type 1 patients with cutaneous neurofibromas (cNF) includes 37 closed-ended questions and 5 open-ended questions on the following domains: patients’ profile (age, gender, family situation, learning difficulties, number of cNF, age at time of first cNF, circumstances around cNF appearance), living with cNF (inconveniences caused by cNF, impact on quality of life, medical monitoring, access to specialized medical center), patients’ satisfaction with cNF management (access to and satisfaction with laser and/or surgical treatments, other medications used), and patients’ expectations in terms of future treatments (best galenic form, effectiveness criteria, risk benefit balance, acceptable period of treatment, acceptable frequency of administration). Three additional questions are slipped into the questionnaire as a comprehension test (checking test, ranking test, and table test). [file 13023_2019_1265_MOESM1_ESM.pdf]

Patient survey for online Carenity patient community

Project Neurofibromatosis type 1 with cutaneous neurofibromas

**Subjects**	The objective of this survey is to have patients' feedback about:<br>- feelings about the disease burden<br>- feelings about current standard of care<br>- expectations in terms of future treatment
**Structure**	A. RESPONDENTS' PROFILE<br>B. LIVING WITH CUTANEOUS NEUROFIBROMAS<br>C. PATIENTS' SATISFACTION WITH CUTANEOUS NEUROFRIBROMAS MANAGEMENT<br>D. PATIENTS' EXPECTATIONS IN TERMS OF FUTURE TREATMENTS
**Number of questions**	42 questions

Introduction message:

Hello,

We are pleased to invite you to participate in our international survey which aims at understanding your everyday life with Neurofibromatosis Type 1, your satisfaction with your current medical care, your expectations regarding future medications for treating cutaneous neurofibromas.

This survey is part of an international project that Carenity is conducting in partnership with the Research & Development Department of a pharmaceutical company.

Your participation in this survey will allow patients' voice to be heard!

Your answers will be secured, aggregated (no individual answers will be shared) and confidential. You can take a break from the questionnaire at any moment and resume it whenever you wish to.

Thank you in advance for your contribution.

The Carenity team

Message to be displayed for the patients: To ensure that you stay focused all through the questionnaire, we slipped some extra questions into the survey. Don't be surprised!

1. You are:

(1 answer possible)

- ☐ A patient affected with Neurofibromatosis Type 1 (NF1)
- ☐ A caregiver to a patient affected with Neurofibromatosis Type 1 (NF1) ***[END OF SURVEY]***
- ☐ Other [please, specify] ***[END OF SURVEY]***

2. **Your year of birth:**

☐ (yyyy) **[END OF SURVEY if age <18 years old]**

3. **You are:**

(1 answer possible)

- ☐ A man
- ☐ A woman

4. **Do you have children?**

(1 answer possible)

- ☐ Yes
- ☐ No **>Q6**

5. **How many children do you have?**

☐ (numerical box)

6. **Have you ever had learning difficulties caused by neurofibromatosis type 1?**

(1 answer possible)

- ☐ Yes
- ☐ No

7. **Do you have cutaneous neurofibromas?**

Cutaneous neurofibromas are benign skin growths that resemble the ones shown in the pictures below.

(1 answer possible)

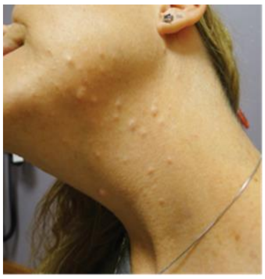
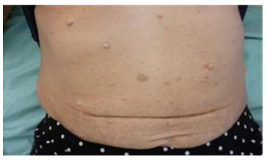
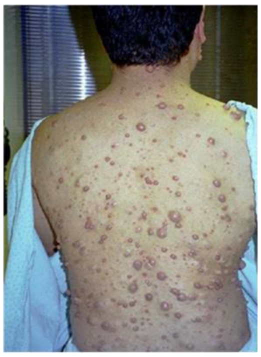
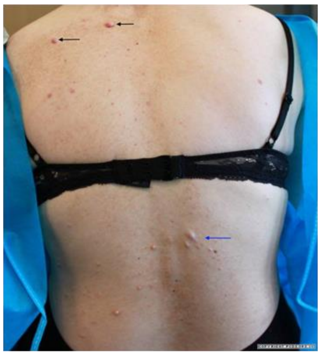
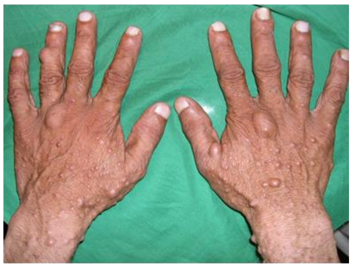

- ☐ Yes
- ☐ No ***[END OF SURVEY]***
- ☐ I don't know ***[END OF SURVEY]]***

1/3. Please check every number higher than 5.

(Several answers possible)

- ☐ 0
- ☐ 2
- ☐ 4
- ☐ 7
- ☐ 8
- ☐ 11

8. Approximately, how many cutaneous neurofibromas have you got on your whole body?

(If you do not know the exact number, please give us your best estimate.)

☐ (numerical box)

9. Approximately, how many cutaneous neurofibromas have you got on the visible parts of your body (face, hands, forearm, neck, forearm etc.)?

(If you do not know the exact number, please give us your best estimate. If you have no cutaneous neurofibromas on the visible parts of your body, please enter "0".)

☐ (numerical box)

10. In total, approximately how many cutaneous neurofibromas larger than 1 centimeter have you got?

(1 answer possible)

- ☐ Less than 10
- ☐ From 10 to 50
- ☐ From 50 to 100
- ☐ More than 100
- ☐ None

11. In which circumstances did you notice the appearance or the growth (increase in size or appearance of new one) of cutaneous neurofibromas?

(Several answers possible)

- ☐ Pregnancy
- ☐ Puberty
- ☐ Skin incision or injury
- ☐ Piercing
- ☐ Sun exposure
- ☐ Hormonal treatments (contraceptives, menopause treatments)
- ☐ Stress
- ☐ Other *[Please, specify]*
- ☐ My cutaneous neurofibromas can appear without any specific reasons

12. How old were you when the first cutaneous neurofibromas appeared?

☐ Age box

13. **What bothers you most about your cutaneous neurofibromas? Please, rank from the most important to the least important.**
Please, rank the following items according to their importance: 1 = this aspect bothers me most, 8 = this aspect bothers me least.

- ☐ Pain caused by cutaneous neurofibromas
- ☐ Itching caused by cutaneous neurofibromas
- ☐ The size of cutaneous neurofibromas
- ☐ The number of cutaneous neurofibromas
- ☐ The localisation of cutaneous neurofibromas
- ☐ The possible evolution of the disease severity
- ☐ Unaesthetic aspect and visibility of cutaneous neurofibromas
- ☐ Nothing really bothers me in regards to cutaneous neurofibromas

14. **Is there anything else that bothers you about your cutaneous neurofibromas?**
Please provide a detailed answer

Text box

15. **What is the negative impact of cutaneous neurofibromas on your daily life?**
Give a score from 0 to 10 (0= no negative impact, 10 = considerable negative impact)
(1 answer per line)

	0 = no negative impact	1	2	3	4	5	6	7	8	9	10 = considerable negative impact	Not concerned
Professional life (finding a job, getting promoted, etc.)	o	o	o	o	o	o	o	o	o	o	o	o
Social life (approaching people, going out, the way others look at you, being isolated, etc.)	o	o	o	o	o	o	o	o	o	o	o	o
Family life (outdoor family activities, mockery suffered by your entourage etc.)	o	o	o	o	o	o	o	o	o	o	o	o
Private life (sexuality, dates, etc.)	o	o	o	o	o	o	o	o	o	o	o	o
Desire to have children	o	o	o	o	o	o	o	o	o	o	o	o
Everyday mood (self-esteem, self-confidence, depression, mood swings, etc.)	o	o	o	o	o	o	o	o	o	o	o	o
Money spent to manage	o	o	o	o	o	o	o	o	o	o	o	o

cutaneous neurofibromas (cost of medications, medical appointments, etc.)												
Daily life in general	o	o	o	o	o	o	o	o	o	o	o	o

16. How often are you monitored by the following doctors for your neurofibromatosis type 1?
(1 answer per line)

	Every month	Every 3 months	Every 6 months	Every year	Less than yearly	I am not monitored by this doctor
General practitioner	o	o	o	o	o	o
Dermatologist	o	o	o	o	o	o
Ophthalmologist	o	o	o	o	o	o
Neurologist	o	o	o	o	o	o
Genetic counsellor	o	o	o	o	o	o
Orthopedist	o	o	o	o	o	o

Q16bis. Are you regularly seen by a neurofibromatosis specialist in a specialised medical center (such as the reference center)?
(1 answer)
☐ Yes
☐ No

17. Have you ever had cutaneous neurofibromas removed by laser sessions*?
(1 answer possible)
**The CO2 laser uses light beams to remove cutaneous neurofibromas.*
☐ Yes
☐ No **> Q22**

18. How many laser sessions have you had?
(If you do not recall the exact number, please give us your best estimate.)
☐ (numerical box)

19. Approximately, how many cutaneous neurofibromas were removed <u>per laser session</u>?
(Number of removed cutaneous neurofibromas can vary from one session to another (depending on the treated area), please provide an average.)
☐ (numerical box)

20. Approximately, how many <u>visible</u> cutaneous neurofibromas (on the face, hands, neck, forearm, etc.) were removed <u>per laser session?</u>
(Number of removed cutaneous neurofibromas can vary from one session to another (depending on the treated area), please provide an average.)

☐ (numerical box)

21. To what extent are you satisfied with the laser sessions during which your cutaneous neurofibromas were removed?

Give a score from 0 to 10 (0= not satisfied at all, 10 = very satisfied)

(1 answer per line)

	0 = not at all satisfied	1	2	3	4	5	6	7	8	9	10 =very satisfied	Don't know / Not concerned
General satisfaction with the laser sessions	o	o	o	o	o	o	o	o	o	o	o	o
Accessibility of a health centre offering laser sessions	o	o	o	o	o	o	o	o	o	o	o	o
Accessibility of laser sessions under general anaesthesia	o	o	o	o	o	o	o	o	o	o	o	o
Frequency of laser sessions	o	o	o	o	o	o	o	o	o	o	o	o
Disagreement during the laser session: burning sensations, anesthesia, duration of the operation	o	o	o	o	o	o	o	o	o	o	o	o
Number of removed cutaneous neurofibromas during one laser session	o	o	o	o	o	o	o	o	o	o	o	o
Effect on small cutaneous neurofibromas	o	o	o	o	o	o	o	o	o	o	o	o
Effect on large cutaneous neurofibromas	o	o	o	o	o	o	o	o	o	o	o	o
Scars after the removal of cutaneous neurofibromas	o	o	o	o	o	o	o	o	o	o	o	o
Regrowth of cutaneous neurofibromas after sessions	o	o	o	o	o	o	o	o	o	o	o	o
Burden of necessary care after the laser session (healing time, managing pain, etc.)	o	o	o	o	o	o	o	o	o	o	o	o
Cost of the laser sessions	o	o	o	o	o	o	o	o	o	o	o	o

2/3. Please rank these objects from the smallest to the biggest one.

1 = the biggest item, 5 = the smallest item.

- ☐ A very small one
- ☐ A small one
- ☐ A quite big one
- ☐ A big one
- ☐ A very big one

22. **Have you ever had cutaneous neurofibromas removed by surgeries*?**

(1 answer possible)

** The surgeon cuts the cutaneous neurofibromas out of the body before resealing the skin.*

☐ Yes

☐ No > Q27

23. **How many surgeries have you had?**

(If you do not recall the exact number, please give us your best estimate.)

☐ (numerical box)

24. **Approximately, how many cutaneous neurofibromas were removed per surgery?**

(Number of removed cutaneous neurofibromas can vary from one surgery to another (depending on the treated area), please provide an average.)

☐ (numerical box)

25. **Approximately, how many visible cutaneous neurofibromas (on the face, hands, neck, etc.) were removed per surgery?**

(Number of removed cutaneous neurofibromas can vary from one surgery to another (depending on the treated area), please provide an average.)

☐ (numerical box)

26. **To what extent are you satisfied with the surgeries during which your cutaneous neurofibromatomas were removed?**

Give a score from 0 to 10 (0= not satisfied at all, 10 = very satisfied)

(1 answer per line)

	0 = not at all satisfied	1	2	3	4	5	6	7	8	9	10 =very satisfied	Don't know / Not concerned
General satisfaction with the surgeries	o	o	o	o	o	o	o	o	o	o	o	o
Accessibility of a health centre offering surgeries	o	o	o	o	o	o	o	o	o	o	o	o
Frequency of the surgeries	o	o	o	o	o	o	o	o	o	o	o	o
Disagreement during the surgery: anesthesia, pain, duration of the operation	o	o	o	o	o	o	o	o	o	o	o	o
Number of removed cutaneous neurofibromas during one surgery	o	o	o	o	o	o	o	o	o	o	o	o
Effect on small cutaneous neurofibromas	o	o	o	o	o	o	o	o	o	o	o	o
Effect on large cutaneous neurofibromas	o	o	o	o	o	o	o	o	o	o	o	o

Scars after the removal of cutaneous neurofibromas	o	o	o	o	o	o	o	o	o	o	o	o
Regrowth of cutaneous neurofibromas after surgeries	o	o	o	o	o	o	o	o	o	o	o	o
Burden of necessary care after the surgery (healing time, changing bandages, managing pain, etc.)	o	o	o	o	o	o	o	o	o	o	o	o
Cost of the surgery	o	o	o	o	o	o	o	o	o	o	o	o

[Q27 is only for respondents who answer Yes in Q17 and/or Yes in Q22]

27. Following your operations (laser sessions or surgeries), have new cutaneous neurofibromas appeared?

(Several answers possible)

- ☐ Yes, exactly on the same spots where I had cutaneous neurofibromas before the operations
- ☐ Yes, around the areas where I had cutaneous neurofibromas before the operations
- ☐ Yes, but not where I had cutaneous neurofibromas before the operations
- ☐ No, no new cutaneous neurofibromas have appeared since

28. Did you take or have you been taking any other medications (pills, cream, etc.) or did you use any alternative medicines (physiotherapy, acupuncture, homeopathy, etc.) which improved/have improved your cutaneous neurofibromas?

(1 answer possible)

- ☐ Yes
- ☐ No **> Q30**

29. Please tell us which medications (pills, cream, etc.) you took/have been taking or which alternative medicine (physiotherapy, acupuncture, homeopathy, etc.) you used, describe the advantages and the drawbacks for each of them and tell us who recommended them to you.

Text box

30. In your opinion, what are the effectiveness criteria of a future medication for treating cutaneous neurofibromas? Please, rank from the most important to the least important.

1= this criterion is the most important in my view, 6= this criterion is the least important in my view.

- ☐ Block growth of cutaneous neurofibromas
- ☐ Reduce the number of cutaneous neurofibromas
- ☐ Reduce the size of cutaneous neurofibromas
- ☐ Prevent the appearance of cutaneous neurofibromas
- ☐ Prevent the regrowth of cutaneous neurofibromas
- ☐ Reduce the itching / pain of the cutaneous neurofibromas

31. In your opinion, what are other effectiveness criteria of a future medication for managing cutaneous neurofibromas?

Please provide a detailed answer

Text box

32. In your opinion, which aspect is the most important for a future medication for managing cutaneous neurofibromas?

(1 answer possible)

- ☐ A moderately effective but well-tolerated treatment
- ☐ A highly effective treatment with some side effects

3/3. Please check the correct number for each line.

(1 answer per line)

	0	1	2	3	4	5	6	7	8	9	10	Don't know
Check number 2	o	o	o	o	o	o	o	o	o	o	o	O
Check number 4	o	o	o	o	o	o	o	o	o	o	o	O
Check number 7	o	o	o	o	o	o	o	o	o	o	o	O
Check number 0	o	o	o	o	o	o	o	o	o	o	o	o

33. Would you consider this future treatment effective if it could clear a minimum of:

(1 answer possible)

	Not at all effective	Moderatel y effective	Effective	Very effective	Don't know
1 on 10 neurofibromas (10%)	o	o	o	o	o
3 on 10 neurofibromas (30%)	o	o	o	o	o
5 on 10 neurofibromas (50%)	o	o	o	o	o
7 on 10 neurofibromas (70%)	o	o	o	o	o
9 on 10 neurofibromas (90%)	o	o	o	o	o

34. In your opinion, what would be the best form for a future medication?

Please, rank the following form from the best to the worst: 1= this form is the best form in my opinion, 4 = this form is the worst form in my opinion.

- ☐ Cream or ointment
- ☐ Pill or capsule
- ☐ Injection in your cutaneous neurofibromas
- ☐ Implant (a small object inserted under the skin which delivers the medication)

35. **For how long (max.) would you be ready to take this future treatment in the form of *[ANSWER Q34 - first ranked]*?**

(*1 answer possible)*

- ☐ Up to 3 months
- ☐ Up to 6 months
- ☐ Up to 1 year
- ☐ Up to 5 years
- ☐ All my life

36. **How much time (max.) would you be ready to wait before this future medication in the form of *[ANSWER Q34 - first ranked]* have a visible effect on your cutaneous neurofibromas?**

(1 answer possible)

- ☐ Between a week and a month
- ☐ Between 1 and 3 months
- ☐ Between 3 and 6 months
- ☐ Between 6 months and 1 year
- ☐ More than a year

[Q37 is for all forms of treatment except Implant]

37. **You have chosen the following form: *[ANSWER Q34 - first ranked].* What would be the maximum acceptable frequency of administration?**

(1 answer possible)

- ☐ Less than once a month
- ☐ Between once a week and once a month
- ☐ Between once a day and once a week
- ☐ Once a day
- ☐ Twice a day
- ☐ 3 times a day or more

[Q37bis is only for the implant]

Q37bis. You have chosen the following form: *[ANSWER Q34 - first ranked].* What would be the maximum acceptable frequency of renewal?

(1 answer possible)

- ☐ Every month
- ☐ Every 6 months
- ☐ Every year
- ☐ Every 5 years

38. **Would you be ready to pay to take this future medication?**

(1 answer possible)

- ☐ Yes

☐ No > **Q40**

39. What is the maximal price that you would be ready to pay by month to take this future treatment?

Number box

40. In conclusion, what in your opinion would be the best medication to treat cutaneous neurofibromas?

Please provide a detailed answer

Text box
